# Effect of electronic records on mortality among patients in hospital and primary healthcare settings: a systematic review and meta-analyses

**DOI:** 10.3389/fdgth.2024.1377826

**Published:** 2024-06-26

**Authors:** Tariku Nigatu Bogale, Lemma Derseh, Loko Abraham, Herman Willems, Jonathan Metzger, Biruhtesfa Abere, Mesfin Tilaye, Tewodros Hailegeberel, Tadesse Alemu Bekele

**Affiliations:** ^1^ John Snow Research and Training Institute, Inc. (JSI), Addis Ababa, Ethiopia; ^2^ University of Gondar, Gondar, Ethiopia; ^3^ John Snow Research and Training Institute, Inc. (JSI), Boston, MA, United States; ^4^ John Snow Research and Training Institute, Inc. (JSI), Washington, DC, United States; ^5^ United State Agency for International Development, Addis Ababa, Ethiopia

**Keywords:** effect, electronic medical records, electronic health records, electronic records, mortality, systematic review, meta-analyses

## Abstract

**Background:**

Electronic medical records or electronic health records, collectively called electronic records, have significantly transformed the healthcare system and service provision in our world. Despite a number of primary studies on the subject, reports are inconsistent and contradictory about the effects of electronic records on mortality. Therefore, this review examined the effect of electronic records on mortality.

**Methods:**

The review followed the Preferred Reporting Items for Systematic Reviews and Meta-analyses 2020 guideline. Six databases: PubMed, EMBASE, Scopus, CINAHL, Cochrane Library, and Google Scholar, were searched from February 20 to October 25, 2023. Studies that assessed the effect of electronic records on mortality and were published between 1998 and 2022 were included. Joanna Briggs Institute quality appraisal tool was used to assess the methodological quality of the studies. Narrative synthesis was performed to identify patterns across studies. Meta-analysis was conducted using fixed effect and random-effects models to estimate the pooled effect of electronic records on mortality. Funnel plot and Egger's regression test were used to assess for publication bias.

**Results:**

Fifty-four papers were found eligible for the systematic review, of which 42 were included in the meta-analyses. Of the 32 studies that assessed the effect of electronic health record on mortality, eight (25.00%) reported a statistically significant reduction in mortality, 22 (68.75%) did not show a statistically significant difference, and two (6.25%) studies reported an increased risk of mortality. Similarly, among the 22 studies that determined the effect of electronic medical record on mortality, 12 (54.55%) reported a statistically significant reduction in mortality, and ten (45.45%) studies didn't show a statistically significant difference. The fixed effect and random effects on mortality were OR = 0.95 (95% CI: 0.93–0.97) and OR = 0.94 (95% CI: 0.89–0.99), respectively. The associated I-squared was 61.5%. Statistical tests indicated that there was no significant publication bias among the studies included in the meta-analysis.

**Conclusion:**

Despite some heterogeneity among the studies, the review indicated that the implementation of electronic records in inpatient, specialized and intensive care units, and primary healthcare facilities seems to result in a statistically significant reduction in mortality. Maturity level and specific features may have played important roles.

**Systematic Review Registration:**

PROSPERO (CRD42023437257).

## Background

Electronic records (electronic medical records (EMR) and electronic health records (EHR)) are digital records of patients' health and services received. EMR contains medical and treatment information about a patient within one healthcare organization and is used by health service providers for diagnosis and treatment purposes, while EHR contains more comprehensive health information about a patient from multiple health providers. Unlike EMR, EHR is designed to be shared across different healthcare settings, allowing for seamless communication and coordination of care between providers. Both EHR and EMR are computerized collections of patient health information, including a patient's demographic information, medications, medical history, pharmaceutical orders, vital signs, laboratory results, radiological reports, allergies, immunizations, and visits. However, EHR contains a wider range of features and tools than EMR, such as clinical decision support, patient portals, electronic prescribing, lab ordering, telehealth, and interoperability. Interoperability helps different systems access and share data with each other, allowing a patient's medical information to move with them to specialists, laboratories, imaging centers, emergency rooms, and pharmacies, both locally and nationally (1, 2).

Both EMR and EHR have significantly transformed the healthcare system and service provision in our world. Information technologies are increasingly becoming crucial in the provision of healthcare services and are successfully handling the health issues and challenges that physicians and other healthcare professionals are facing (2). These technologies automate the examination, medication, and ordering procedures in healthcare facilities, ensuring consistency and readability (2, 3).

Despite numerous published studies on the effect of electronic records on mortality, reports are inconsistent and contradictory. In this context, though the majority of studies (4, 5) reported a reduction in mortality after use of electronic records across ages and causes, some studies reported no significant effect on mortality or an increase in mortality (1, 6). However, due to the influence of various factors on patient outcomes, the direct effect of electronic records may not be easily determined (7). The inconsistencies in the findings of the studies may be related to the complexity of relationships between those factors. When such inconsistent and contradictory findings are reported, a systematic review and meta-analysis can help identify the causes of inconsistencies and contradictions. Studies also treated EHR and EMR as the same. In addition, prior systematic reviews focused on a particular intervention, setting and/or population. Therefore, this systematic review and meta-analysis was designed to understand the effect of EMR and/or EHR on mortality in inpatient, specialized and intensive care units, and in primary healthcare settings, all ages and causes, to help inform current and future scale-up efforts, program directions, and investments.

## Methods

### Protocol and registration

The Preferred Reporting Items for Systematic Reviews and Meta-analyses (PRISMA) 2020 guidelines was followed in reporting this review. The protocol has been registered at PROSPERO with a registration number of CRD42023437257.

### Search strategy

The search strategies were developed considering the Population, Intervention, Control, and Outcome (PICO) mnemonics (8). Keywords and Medical Subject Headings (MeSH) were generated for these components and were combined with Boolean Operators to improve the sensitivity and specificity of the search. First, the authors agreed on the list of keywords and MeSH terms as well as on databases to be reviewed. Then, the terms were combined according to the advanced search methods of the respective databases. PubMed (including Ovid Medline), EMBASE, Scopus, CINAHL, and Cochrane Library databases, and Google Scholar (for grey literature) were searched from February 20 to October 25, 2023. To capture relevant primary studies that were not accessed through the search engine, citation tracking through snowballing and hand searching were applied based on the bibliographies of the reviewed primary studies.

### Eligibility criteria

Generally, the studies were considered eligible if they fulfilled the PICO (Population, Intervention or Exposure, Comparison and Outcome) criteria. Accordingly, the study population were patients of any age, sex, and illness severity and type. In addition, there was no restriction on type of health service providers, health facilities (any type and level), geographies, and year of publication. The intervention was EMR or EHR. Patients in health institutions where EMR or EHR was not implemented were considered control groups. The outcome of interest was mortality regardless of cause.

Experimental, quasi-experimental and observational studies published in English between 1998 and 2022 were included in the review. Studies that did not have both the intervention (exposure) and control (comparison) groups were excluded from the review. Studies must have effect sizes such as odds ratio (OR), relative risk (RR) or attributable difference (AD), or there should be inputs from the two groups that would help us to generate the effect sizes for inclusion into the review. These inputs include sample size and proportion in each group and a 95% confidence interval (CIs) for effect size estimates and *p*-values. In addition, a narrative synthesis of the included studies was included into the review.

### Risk of bias (quality) assessment

The Joanna Briggs Institute (JBI) SUMARI system was used to independently assess the quality of the primary studies by two reviewers (LD and TA) (9). The reviewers appraised the studies using the quality appraisal tool for each study design. Any disagreements between the reviewers were resolved through discussion with TN.

### Data extraction

A data extraction template was developed by LD and reviewed by TN and TA. Then, the data were extracted from all eligible primary studies by LD and TA independently, using the data extraction checklist for experimental and observational studies. Any disagreements were resolved by TN.

Information extracted included country of study, year of publication, study period, study setting, study design, sample size, mortality measures reported, intervention characteristics (target population, duration or maturity and frequency), and the intervention type (EMR or EHR) associated with the outcome of interest.

### Data analysis

The extracted data were cleaned by LD, TN, and TA for further analysis. Descriptive analysis was carried out to summarize the studies included in the review. Mortality was the outcome measure that was reported consistently across multiple individual studies and used for the meta-analysis. Stata version 11 was used for analysis. OR was estimated to calculate the pooled estimates of the effect of EMR or EHR on mortality. Subgroup analyses were conducted to control the effect of confounding variables. When multiple (≥2) individual studies targeting the same population reported on the same outcome measures, the results were pooled in Stata Software (version 14) using the package “metan” for fixed and random-effects meta-analysis. I^2^ statistics were used to assess for heterogeneity among primary studies, and sensitivity analysis was done to assess the robustness of the results. Funnel plot and Egger's regression test were used to check for publication bias. Forest plots were used to report individual study outcomes, pooled effect sizes, 95% confidence intervals, and *I*^2^ values.

### Operational definitions

Based on the Healthcare Information and Management Systems Society EMR Adoption Model (10), EMR applications can be grouped into three categories, which can be considered the “stage of EMR implementation” based on their maturity level.

• Health facilities that have started implementing the three core ancillary department information systems (i.e., pharmacy, laboratory, and radiology) and a clinical data repository are considered “EMR stage 1”. Automation of the patient record, easier departmental and interdepartmental communication, and improved access to clinical data are the functional characteristics of EMR stage 1.
• Health facilities in “EMR stage 2” have begun implementing nursing documentation and electronic medication administration records in addition to all EMR stage 1 applications. EMR stage 2 functionality is characterized by automation of nursing workflow processes, including clinical documentation and electronic recording of medication administration.
• Health facilities at “EMR stage 3” have implemented all EMR stage 1 and stage 2 applications and have started implementation of Clinical Decision Support (CDS) and Computerized Physician Order Entry (CPOE). EMR stage 3 functionality is characterized by automation of clinical decision processes, including order entry management and support of clinical decision making.

Meaningful Use (MU) of EHR is the utilization of a certified EHR system to improve quality, safety, efficiency, and reduce health disparities; improve care coordination; improve population and public health; engage patients and their families in their own health care; and ensure that patient privacy and security are maintained according to the Health Insurance Portability and Accountability Act Privacy Rule (11). There are three basic components of meaningful use:

1. The use of a certified EHR in a meaningful manner.
2. The electronic exchange of health information to improve the quality of healthcare and,
3. The use of certified EHR technology to submit clinical quality and other measures.

These basic components are implemented gradually through the three stages of EMR implementation.

## Results

### Search process and results

**Figure 1:**
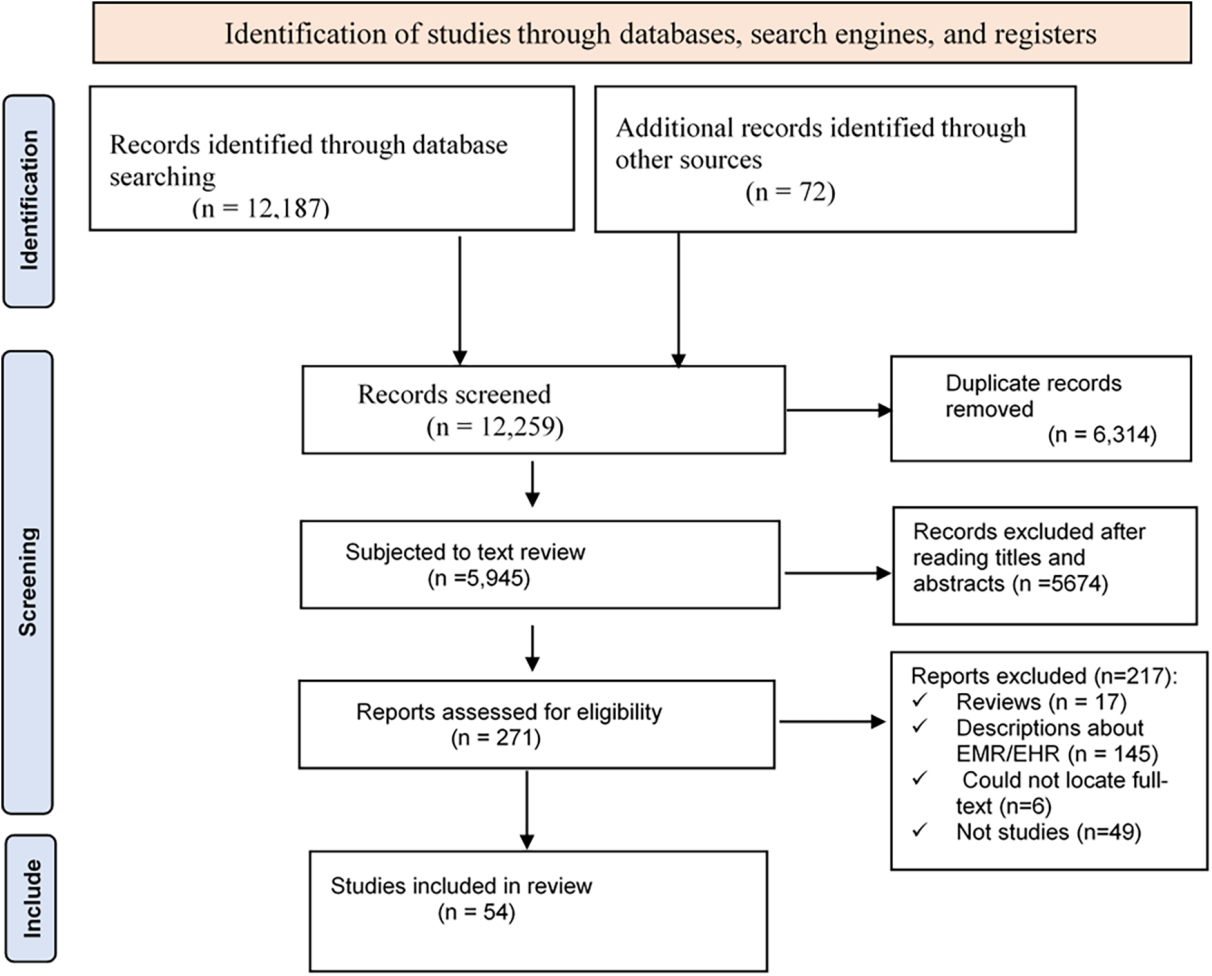
PRISMA diagram showing the identification, screening and inclusion of primary studies.

In the search process, a total of 12,187 studies were identified (PubMed (829), Scopus (1,735), Embase (3,568), CINAHL (1,013), Cochrane (2,182), and Google Scholar (2,860)). Seventy-two additional articles were identified through citation tracking, which brought the total to 12,259. Using endnote, 6,314 duplicate articles were excluded, which reduced the number of articles to 5,945. After reading the title and abstract of the remaining papers, 5,674 were excluded as they were not found eligible for the review. Of the remaining 271 articles, 217 were excluded for different reasons, resulting in 54 eligible articles (Figure 1).

### Key features of papers included

Overall, there were 54 studies published in 25 years from 1998 to 2022 (1, 4–6, 12–61). The majority, 36 (66.67%), of the articles were from the USA (1, 4–6, 12–21, 24–27, 29–31, 33, 35, 37, 38, 40, 41, 43–46, 48, 50, 51, 55, 57, 59). The remaining articles were from Australia, 4 (7.41%) (32, 42, 52, 53), South Korea, 3 (5.56%) (22, 28, 36), UK, 2 (3.70%) (58, 61), England, 2 (3.70%) (1, 60), Taiwan, 1 (1.85%) (22), China, 1 (1.85%) (34), Saudi Arabia, 1 (1.85%) (39), France, 1 (1.85%) (47), Canada, 1 (1.85%) (54), Belgium, 1 (1.85%) (56), and in three countries (Israel, Germany, and Italy), 1 (1.85%) (49) (Table 1). Of those studies that assessed mortality, 7 (12.96%) studies assessed only mortality or took mortality as their primary objective (1, 4, 24, 32, 33, 40, 41), and 47 (87.04%) studies assessed both mortality and service quality (6, 12–23, 25–31, 34–39, 42–61).

**Table 1:** Frequency of studies with specific intervention types and its features by country.

Intervention with specific feature	Number (percent) of studies	Number (percent) of studies on		Country (number)
		Mortality	Service quality and mortality	
EHR with alert	11 (20.37)	2 (3.70)	9 (16.67)	USA (9), Saudi Arabia (1), and South Korea (1)
EHR with website	11 (20.37)	3 (5.56)	8 (14.81)	USA (6), Australia (3), Canada (1), and three countries (Israel, Germany, and Italy) (1)
EHR with reminder	1 (1.85)	–	1 (1.85)	USA (1)
EHR with e-mail	1 (1.85)	–	1 (1.85)	USA (1)
EHR with any combination of features	1 (1.85)	1 (1.85)	–	USA (1)
EHR with features not clearly stated	5 (9.26)	–	5 (9.26)	USA (4) and England (1)
EMR with alert	11 (20.37)	–	11 (20.37)	USA (6), Belgium (1), UK (2), England (1), and South Korea (1)
EMR with website	3 (5.56)		3 (5.56)	USA (2) and France (1)
EMR with reminder	1 (1.85)		1 (1.85)	USA (1)
EMR with features not clearly stated	9 (16.67)***	1 (1.85)	8 (14.81)	USA (5), Australia (1), China (1), South Korea (1), and Taiwan (1)
Overall	54 (100)	7 (12.96)	47 (87.03)	–

Of the 54 studies included in the review, 26 (48.15%) assessed the effect of EHR on both mortality and service quality (5, 6, 12, 13, 16–18, 20, 25, 28, 29, 31, 35, 37, 39, 42–47, 49, 51–54), 21 (38.89%) studies assessed the effect of EMR on both mortality and service quality (14, 15, 19, 21–23, 26, 27, 30, 34, 36, 38, 48, 50, 55–61), six (11.11%) studies assessed the effect of EHR on mortality (1, 4, 32, 33, 40, 41), and one (1.85%) study assessed the effect of EMR on mortality (32).

### Interventions with specific features

The primary studies provided explanations about the specific features embedded within EMR or EHR systems and their effect on mortality and service quality. In this regard, 11 (20.37%) studies used EHR with alert (13, 17, 18, 20, 24, 25, 28, 29, 33, 39, 44), 11 (20.37%) studies used EHR with website (4, 6, 40–43, 45, 49, 52–54), one (1.85%) study used EHR with reminder (37), one (1.85%) study used EHR with E-mail (51), one (1.85%) study used EHR with any combination of these (1), and five (9.26%) studies used EHR with unspecified feature(s) (5, 12, 16, 31, 35). In addition, 11 (20.37%) studies used EMR with alert (19, 26, 27, 36, 50, 55–58, 60, 61), three (5.56%) studies used EMR with website (46, 47, 59), one (1.85%) study used EMR with reminder (48), and 9 (16.67%) studies used EMR with unspecified feature(s) (14, 15, 21–23, 30, 32, 34, 38) (Table 1).

### Study design and risk of bias

The studies used different designs. Accordingly, 23 (42.59%%) studies were retrospective cohort studies (5, 6, 14, 15, 17–19, 21–23, 26, 31, 33–38, 44, 48, 50, 51, 54), 17 (31.48%) pre-post studies (1, 4, 24, 27, 28, 32, 39–43, 45–47, 55, 56, 58), five (9.26%) prospective cohort studies (16, 52, 53, 57, 61), four (7.41%) were RCTs (13, 25, 29, 49), three (5.56%) were cross-sectional studies (12, 20, 59), and two (3.70%) were stepped wedge study designs (30, 60). The risk of bias assessment for the studies revealed that there was no significant concern, though the score of some studies is relatively lower than others. Accordingly, the 28 cohort studies (both retrospective and prospective) scored 7 to 11 points out of 11 questions; the 20 pre-post and stepped wedge studies scored 6 to 9 points out of 9 questions; the four RCT studies scored 12 to 13 points out of 13 questions; and the three cross-sectional studies scored 6 to 7 points out of 8 questions.

### Study settings and participants

The studies were conducted in different settings and among multiple categories of study populations. Thirty five (64.81%) (4, 5, 15–24, 26, 28–37, 40, 43–47, 49, 50, 53, 59–61) of the studies were conducted in hospital settings, and 19 (35.19%) (1, 6, 12–14, 25, 27, 38, 39, 41, 42, 48, 51, 52, 54–58) studies were conducted in academic medical centers or medical clinics. Forty nine (90.74%) (1, 4–6, 12–14, 16–19, 21–23, 25–29, 31–36, 38–61) of the studies included patients, three(5.56%) (15, 20, 37), were among clinicians, and nurses, 1 (1.85%) (30). Similarly, there was one (1.85%) study that was conducted by considering vendors as its study population (24) (Table 2).

**Table 2:** Frequency of studies with specific groups of participants within each study setting.

Study population	Settings in which the participants were studied		Total
	Hospitals	Academic medical centers/clinics	
Patients	30 (55.56)	19 (35.19)	49 (90.74)
Hospitals and clinicians	3 (5.56)	0 (0.00)	3 (5.56)
Nurses	1 (1.85)	0 (0.00)	1 (1.85)
Vendors	1 (1.85)	0 (0.00)	1 (1.85)
Total	35 (64.81)	19 (35.19)	54 (100)

### Effects of EHR or EMR on mortality

We examined the effect of EMR or EHR on mortality. In this regard, after the implementation of EMR or EHR, studies reported three different effects: positive effect (a statistically significant reduction in mortality), inconclusive or similar effect (statistically insignificant effect on mortality), and negative effect (a statistically significant increase in mortality).

Of the 32 studies that assessed the effect of EHR on mortality, nine (28.13%) reported positive effect on mortality (5, 12, 16, 18, 20, 24, 33, 35, 41), 21(65.63%) were inconclusive (4, 6, 13, 17, 25, 28, 31, 37, 39, 40, 42–47, 49, 51–54), and two (6.25%) studies reported negative effect on mortality (1, 29). Similarly, of the 22 studies that determined the effect of EMR intervention on mortality, 14 (63.63%) reported a positive effect on mortality (15, 19, 21–23, 26, 30, 32, 34, 36, 38, 48, 59, 61), and eight (36.36%) studies were inconclusive (14, 27, 50, 55–58, 60) (Table 3).

**Table 3:** Number of studies with specific types of intervention and its effect on mortality.

Type of intervention	Effect on mortality rate			Overall
	Statistically significant reduction in mortality	No statistically significant effect on mortality	Significant increase in mortality	
EMR	14 (63.63)	8 (36.36)	0 (0.00)	22 (100)
EHR	9 (28.13)	21 (65.63)	2 (6.25)	32 (100)
Overall	23 (42.59)	29 (53.70)	2 (3.70)	54 (100)

The two studies that reported negative findings were from the USA. The duration of exposure of the study population to EHR or EMR and the study duration vary across studies (Table 4).

**Table 4:** Key characteristics of studies that reported no effect or negative effects of EHR or EMR on mortality.

PI	Country	Study participants	Outcome variable	Design	Duration of the intervention	Intervention
Perry Wilson. et al.	USA	Adult in-patients with acute kidney injury	Time until acute kidney injury progression^a^ including death	Cluster RCT	Followed for 14 days and the study lasted for 7 years	EHR with alert
Yong Y. Han et al.	USA	Children who are transported for specialized care	Reduction in mortality and medical errors	A pre-post study where data was analyzed retrospectively	Thirteen months before and 5 months after CPOE Implementation	EHR (or CPOE) with alerts and reminder regarding interactions and allergies

^a^ The primary outcome was a composite of inpatient acute kidney injury progression (defined as an increase in acute kidney injury stage), receipt of dialysis, or death within 14 days of randomization.

### Intervention duration and mortality

The duration of EHR or EMR implementation or other similar parameters such as EHR meaningful use (see the definition under “operational definition”) or stages of EMR or EHR implementation were examined against health outcomes. Some studies represent the duration of EMR or EHR implementation. With a longer duration of implementation, better maturity of the systems is expected (21, 33). Generally, higher maturity level of EMR or EHR was associated with improved health outcomes. One study, however, found no substantial improvements in process measures or condition-specific outcomes by duration of EHR use or a hospital's status of meaningful use categorized as stage 1 or stage 2 (37).

Specifically, EHR use that met meaningful use stage 1 criteria showed an insignificant association between EHR and mortality (37). Another study, however, showed about 18–19 percent lower mortality after 2–3 years of EHR implementation. The same study indicated that EMR stage 3 increased complications by about 2.0–3.0 percent but decreased mortality by 3.0–4.2 percent after 2–3 years of implementation (15). Another study also showed that after adjusting for confounding factors, EHRs that attested to meaningful use had a positive impact on mortality rate with an 8% decrease in composites for mortality for selected procedures and 18% decrease in composites for mortality for selected conditions (33).

Health facilities that met the criteria for meaningful use stage 2 had higher mortality rates at baseline that decreased with time of exposure to the intervention. On the other hand, health facilities meeting the criteria for meaningful use stage 1 had no significant differences in mortality rates both at baseline and follow up (37).

### Narrative synthesis

Forty-two studies were included in the meta-analysis; 24 of them reported positive effect of EMR or EHR on mortality (4, 5, 12, 13, 16, 17, 19–21, 23, 30, 35, 38–40, 43, 48, 49, 55–59, 61). However, the effect is inconclusive in 15 studies (4, 13, 17, 27, 30, 38–40, 43, 48, 49, 55–58). Eighteen other studies (1, 6, 14, 18, 25, 27–29, 31, 42, 44, 45, 47, 51–54, 60) reported negative effects of EMR or EHR on mortality. However, only two of these studies (1, 29) reported a statistically significant increase in mortality after EHR intervention. One of these two studies assessed the effect of EHR on mortality, and reported that after the intervention, mortality had increased over threefold change (OR = 3.28; 95% CI: 1.94–5.55) (1), and the other study reported a 15.6% increase in mortality rate in the alert group vs. 8.6% in the usual care group which is almost a twofold change (*p*-value = 0.003) (29).

### Meta-analysis

**Figure 2:**
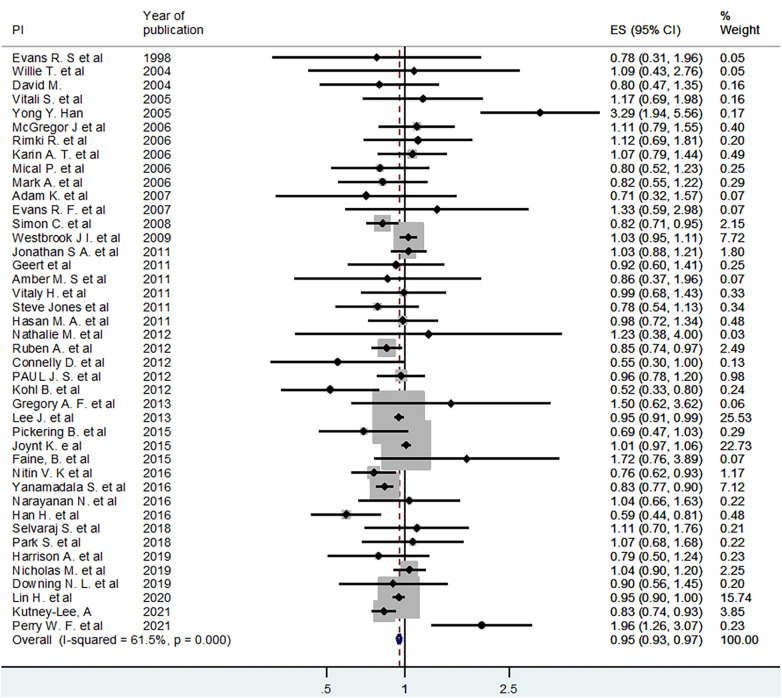
Fixed effect model pooled estimate of 42 studies for the effect of EHR or EMR on mortality.

A meta-analysis was conducted to estimate the pooled (averaged) effect of EMR or EHR on mortality. Out of 54 studies included in the systematic review, 42 studies were eligible for meta-analysis (1, 4–6, 12–14, 16–21, 23, 25, 27–31, 35, 38–40, 42–45, 47–49, 51–61). Accordingly, using the fixed effect model, the pooled or averaged odds ratio (OR) was estimated at 0.95 (95% CI: 0.93–0.97), which was statistically significant. The variation in effect sizes attributable to heterogeneity (I-squared) was 61.5%. This indicates that the use of electronic records (EMR or EHR) reduced mortality by 3–7 percent (Figure 2). When a random effects model was used, the pooled OR was 0.94 (95% CI: 0.89–0.99) (Figure 2).

### Subgroup analyses

**Figure 3:**
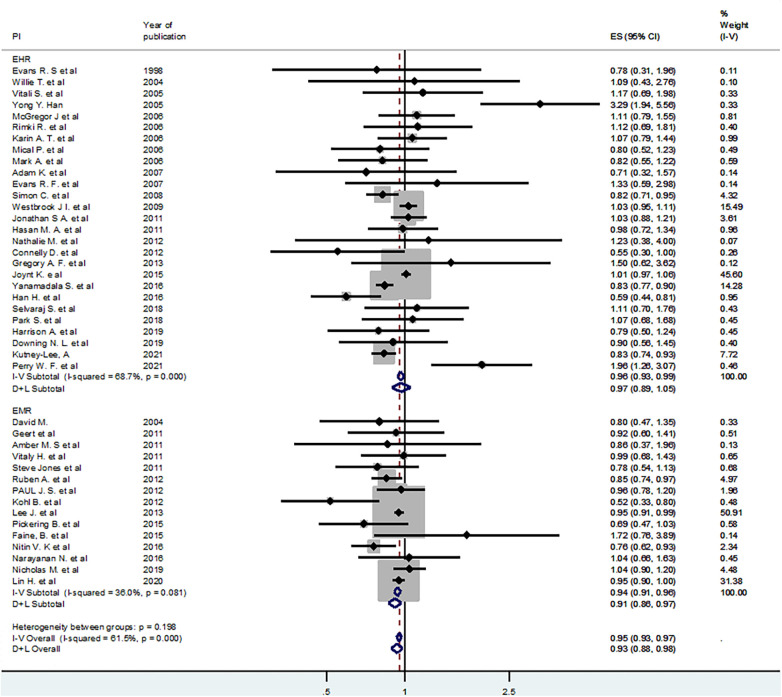
Sub-group analysis by electronic record type (EHR and EMR).

To deal with the observed moderate level of heterogeneity (I-squared = 61.5%), subgroup analyses were conducted. In addition, a random effects model was employed using the DerSimonian and Laird (D + L) approach. First, we used the type of intervention (EHR vs. EMR) as a grouping variable. Using fixed effect and random effects models in the subgroup analysis, the pooled estimates for the effect of EMR on mortality were OR = 0.94 (95% CI: 0.91–0.96) and OR = 0.91 (95% CI: 0.86–0.97), respectively. Similarly, the fixed effect and random effects models pooled estimates for the EHR sub-group were OR = 0.96 (95% CI: 0.93–0.99) and OR = 0.93 (95% CI: 0.88–0.98), respectively. All the pooled estimates were statistically significant, and the value of I-squared for the EMR sub-group dropped largely (Figure 3).

Similarly, subgroup analyses were conducted using region (Western region and Eastern region), type of participants (patients and others such as clinicians and health facilities), intervention features (EHR or EMR with alert, website, or unspecified feature), and study setting (hospital vs. academic medical centers or clinics) (Table 5).

**Table 5:** Subgroup analyses by different characteristics for the effect of EHR or EMR on mortality.

Grouping variable	Group	Fixed effect pooled estimate: OR (95% CI)	Random effect pooled estimate: OR (95% CI)	I-squared
Type of intervention	EHR	0.96 (0.93–0.99)	0.97 (0.89–1.05)	68.7%
	EMR	0.94 (0.91–0.96)	0.91 (0.86–0.97)	36.0%
Participants	Patients	0.96 (0.93–0.98)	0.94 (0.89–0.99)	60.2%
	Others	0.95 (0.93–0.97)	0.93 (0.88–0.98)	0%
Country/region	Western	0.94 (0.92–0.96)	0.92 (0.86–0.98)	65.0%
	Eastern	0.98 (0.94–1.02)	0.98 (0.94–1.02)	0.0%
Setting	Academic	1.03 (0.96–1.09)	1.03 (0.91–1.16)	67.0%
	Hospitals/non-academic	0.94 (0.92–0.96)	0.90 (0.85–0.96)	43.4%
Specific feature of intervention^a^	EHR with alert	0.98 (0.95–1.02)	0.96 (0.88–1.05)	54.5%
	EMR with website	0.98 (0.922–1.05)	0.97 (0.90–1.05)	5.8%
	EHR with unspecified feature	0.82 (0.77–0.88)	0.79 (0.693–0.91)	48.0%
	EMR with alert	0.72 (0.53–0.99)	0.73 (0.369–1.45)	78.9%
	EMR with website	1.23 (0.378–4.00)	1.23 (0.38–4.00)	0.0%
	EMR with unspecified feature	0.95 (0.92–0.98)	0.95 (0.91–0.98)	10.2%

^a^ Four specific features, namely EHR with e-mail, EHR with reminder, EHR with any combination of features, and EMR with reminder, have only one study for each type of feature, resulting in a single group size. Therefore, we have removed these four studies from subgroup analysis.

### Cumulative meta-analysis

**Figure 4:**
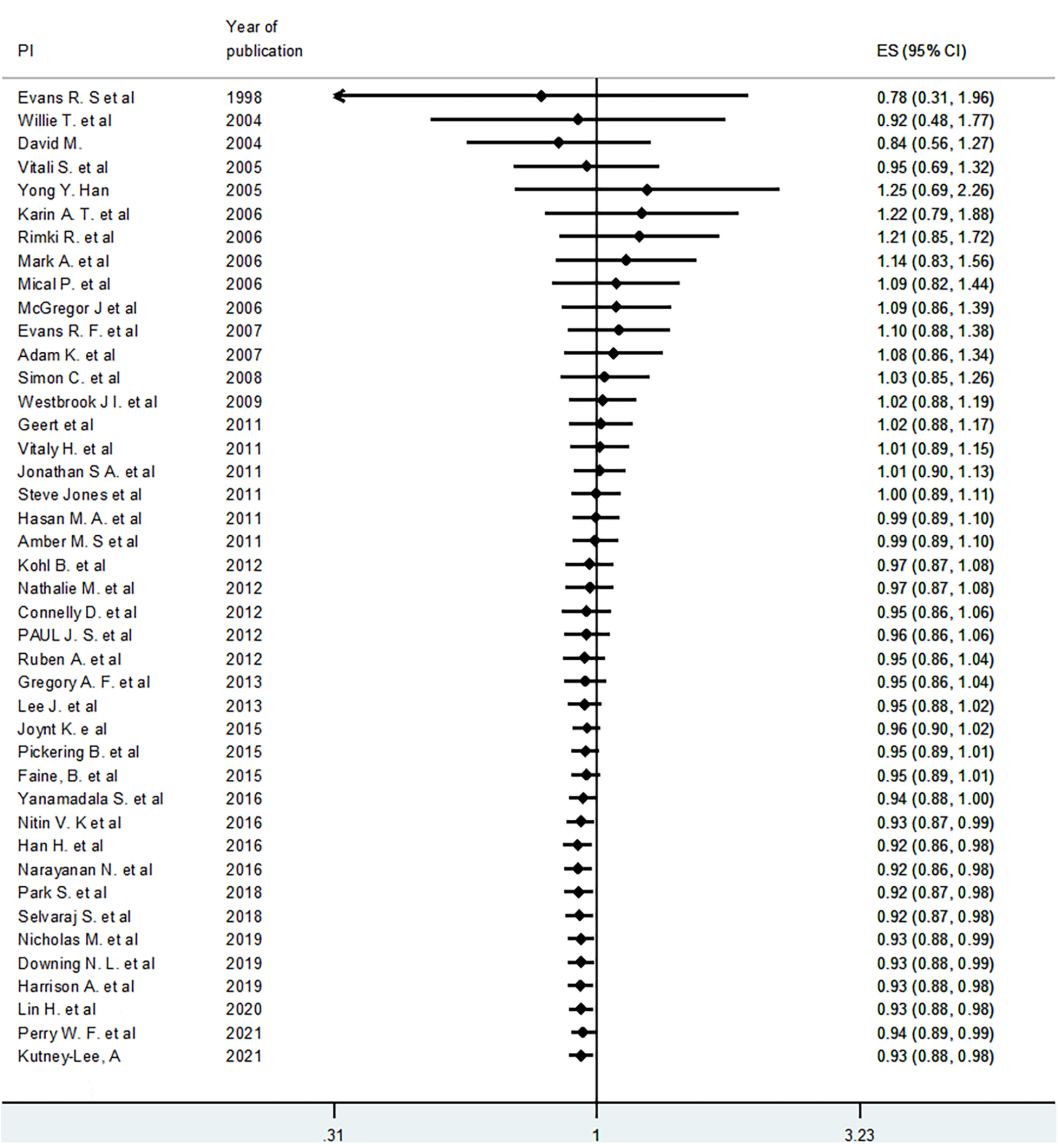
Cumulative meta-analyses for the effect of EHR or EMR on mortality.

To see the pattern of the effect of EHR or EMR on mortality over time, we conducted a cumulative meta-analysis using the random effects model. Though not statistically significant, the cumulative effect was initially (from 1998 to 2005) positive effect on mortality. However, in the next six years (2005–2011), the effect on mortality increasingly became negative, but statistically insignificant. Thereafter, the effect gradually became positive. In other words, as we include more recent studies (later than 2016), the cumulative effects (odds ratio) gradually became less than 1.0 and statistically significant. The 95% CI gets narrower as we proceed to include more recent studies because of an increase in cumulative sample size (Figure 4).

### Sensitivity analysis

**Figure 5:**
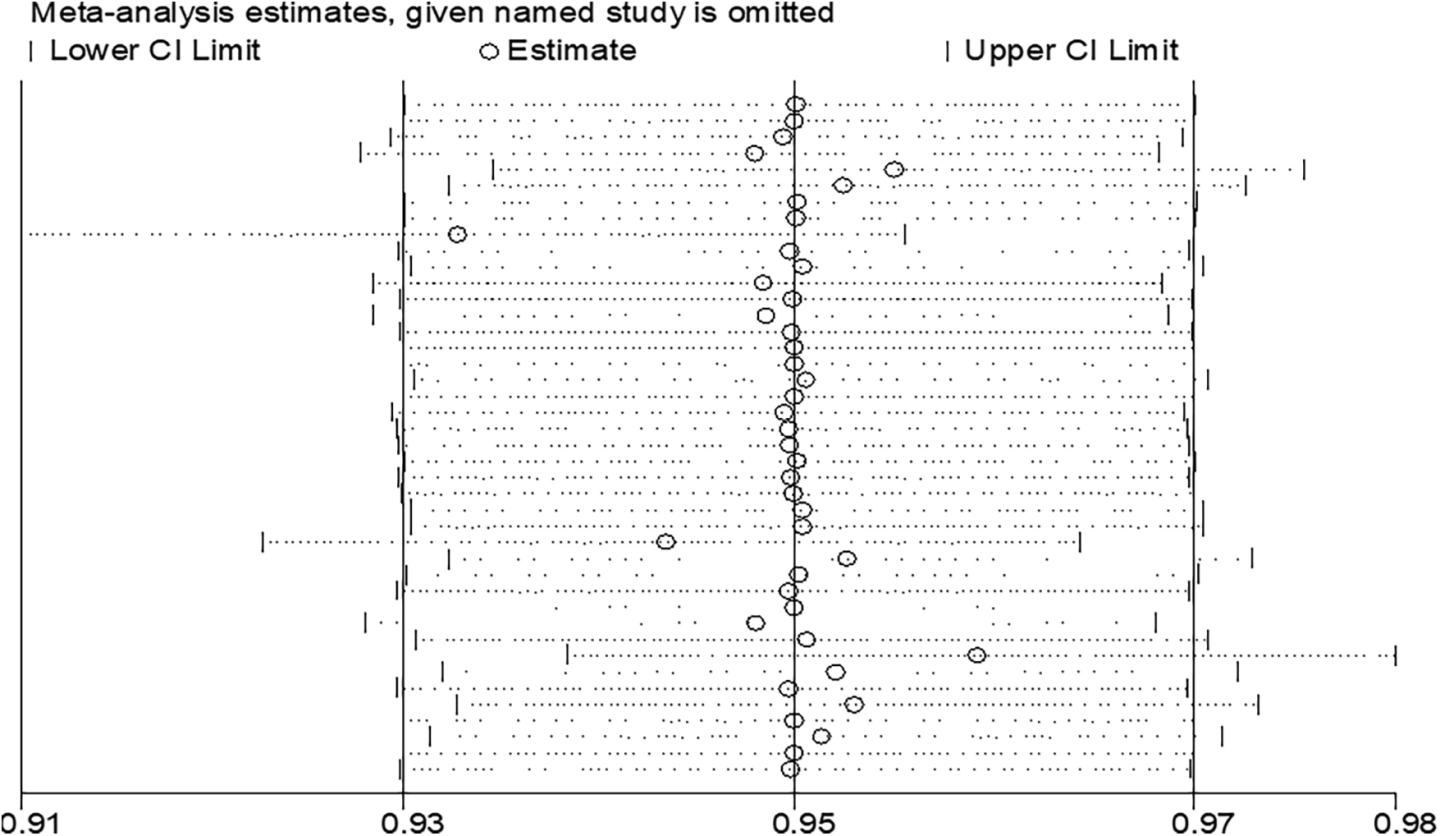
Sensitivity analysis with fixed effect model estimation.

The result of the meta-analyses showed a moderate level of heterogeneity among study estimates. To check whether there are influential or outlier studies that are behind the observed heterogeneity, we conducted sensitivity analysis with a fixed effect model first. However, there was no study such that when it is removed, the pooled estimate of the remaining studies would be out of the overall confidence interval (the 95% CI when all 42 are considered). In other words, when each study is removed, the pooled estimate of the remaining 41 studies would be within the overall (42 studies) confidence interval (Figure 5). This confirms that there was no significantly influential or outlier study.

### Publications bias

In addition to heterogeneity, the other concern when conducting meta-analyses is publication bias or small study effect. This concern is related to the fact that many journals show less interest in publishing small studies. At the same time, small studies have a higher chance of not being statistically significant. As a result, such small studies (unless they have a very strong effect) can be excluded from publication or the web. If they are not published, they would not be accessed by researchers and hence could not be included in systematic reviews and meta-analyses. To minimize this concern, we included unpublished results during literature search from the different databases and other sources. In addition, we conducted both subjective (funnel plot) and objective (Eager's Test) techniques to check for the presence of publication bias so that appropriate measures could be taken accordingly.

**Figure 6:**
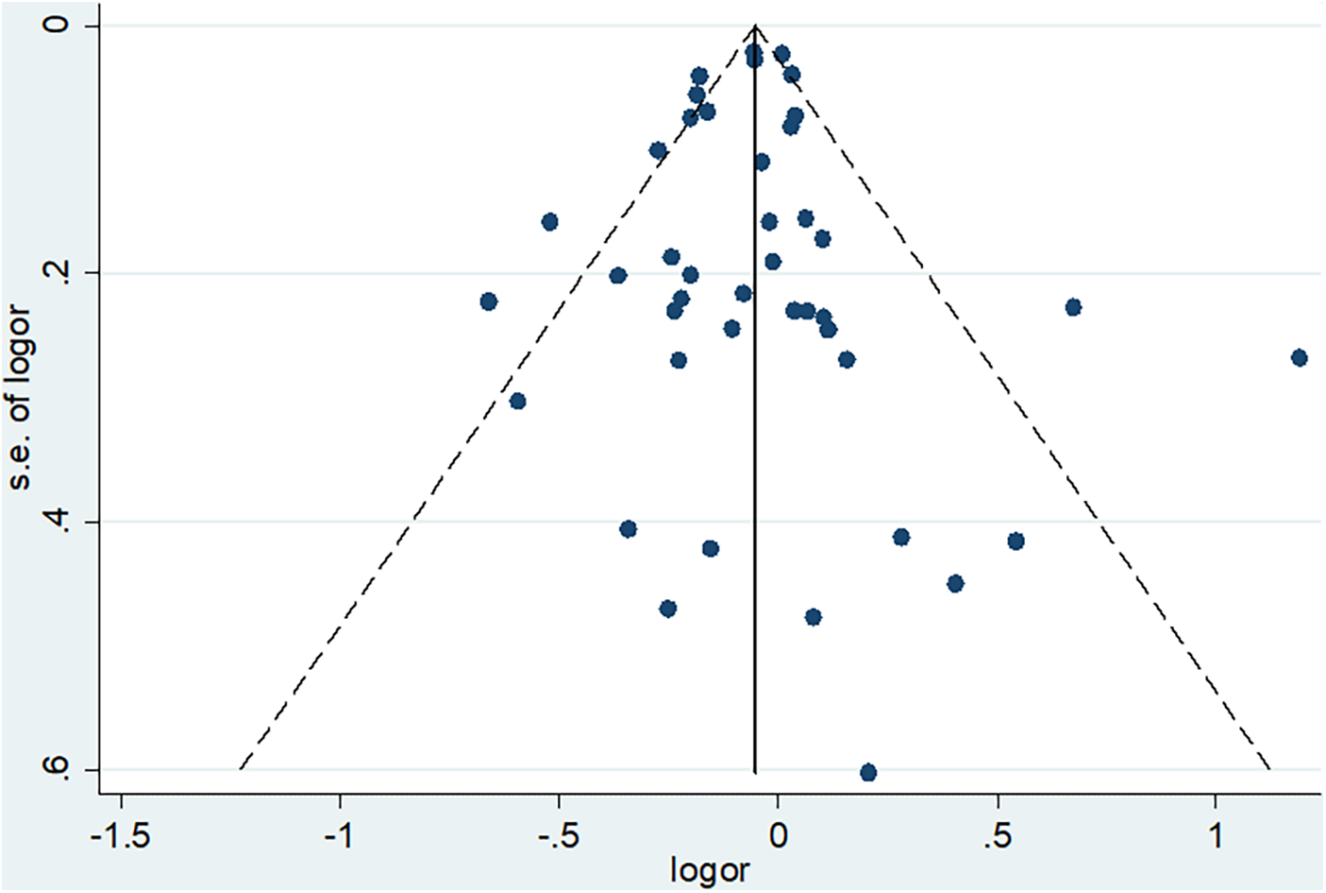
Funnel plot to check publication bias subjectively.

The funnel plot indicates that publication bias may not be a concern in our studies. Even though we have one small study (at the bottom), which is not considerably deviating from the center, it is hard for it to bias the pooled estimate significantly. The other studies are distributed almost symmetrically at the middle or top of the funnel plot, confirming that exclusion from publication of studies with a large sample size (high power of study) is not a concern (Figure 6).

To support the finding from the subjective technique (funnel plot), Eager's test was conducted and confirmed that it is not statistically significant (*p*-value = 0.794). Therefore, our set of studies that reported mortality are not affected by publication bias, and thus there is no need to conduct subsequent measures like Trim and Fill analysis.

## Discussion

This systematic review and meta-analysis determined the effect of electronic records on mortality in inpatient, specialized and intensive care units, and primary healthcare settings. The studies included in this review determined the effect of a particular feature of EMR or EHR, such as computerized physician order entry (CPOE), clinical decision support (CDS) systems like clinical alerts, telemedicine, screening and surveillance systems, or a combination of any of these, on mortality.

The review showed that the majority of the studies that assessed the effect of electronic records on mortality were from the USA. Part of the reason for this could be the exclusion of studies that were published in languages other than English. On average, there was a five percent reduction in mortality associated with the implementation of EMR or EHR. For this pooled estimate, the associated level of heterogeneity among studies may not be a concern as it (I-squared = 61.5%) can be considered moderate (62). Our assessment also showed that publication bias may not be a concern. In attempts made to deal with the observed moderate level of heterogeneity using subgroup analyses, it was further minimized in some groups.

In this review, the overall pooled odds ratio for the effect of EHR or EMR on patient mortality using a fixed effect model was estimated at OR = 0.95 (95% CI: 0.93–0.97). From the test of heterogeneity, we understood that there was a moderate (I-squared = 61.5%) variation among the effect sizes of the studies. Using a random effects model, the corresponding estimate was OR = 0.94 (95% CI: 0.89–0.99). Unlike that of the fixed effect model estimate, the random effects model's pooled estimate has a wide confidence interval, and this could reveal the presence of extra variation due to clustering or variation across studies.

The factors that contribute to the observed moderate level of heterogeneity among studies could be attributed to different characteristics that influence the effective use of electronic records. Thus, one important factor that could affect the magnitude of the effect of electronic records on health outcomes, including mortality, is the type of features embedded and the maturity level of EHR or EMR. These factors could be grouped under five categories including technology, people, organization, resources, and policy (63). Three studies under this review also discussed that the maturation or stages of EMR or EHR implementation play a significant role in improving health outcomes (23, 31, 35).

Evidence revealed that at the earlier stage of electronic records implementation, it might result in insignificant improvement or even negative effect on mortality. However, as the implementation process goes on, it gradually has a positive effect. In the more advanced stages of the EHR or EMR implementation, especially stages 2 and 3, interoperability may not be a concern as the system integration improves over time. One explanation for the progressively improving positive effect of electronic health records on mortality may be related to the fact that health workers could become more familiar with the technical aspects of the technology and improvement in skills. This could in turn improve the timeliness of service provision and the quality of healthcare, which results in improved health outcomes, including mortality.

As indicated in the subgroup analysis, another reason for the high heterogeneity among effect sizes of studies could be the variation in participants, regions, and settings in which the studies were conducted. The heterogeneity of estimates for studies conducted in the “Eastern” region of the globe is none, implying that one reason influencing the effect of electronic records on mortality may be related to the variation in context among regions. Similarly, the zero heterogeneity among studies on participants other than patients indicates that the nature of study units could be another source of variation. However, study units can be defined in many ways, such as the type of health problem resulting in hospitalization (e.g., kidney disease patients (28, 29), surgical patients (17, 20), heart failure patients (12, 31), sepsis patients (26, 27), patients with stroke (18), and health workers (30), etc.).

A systematic review reported that, in addition to technology, other characteristics such as people, organization, resources, and policy can influence the chance of reaching maturity level of EMR or EHR utilization. The challenge is that the characteristics of study units and other factors that could influence the effect of health records on mortality are usually uncontrolled or unmeasured by the primary studies, which makes further analysis, including subgroup analysis, difficult. As a result, it would be impossible to report pooled estimates with the homogeneity of estimates from primary studies.

### Limitations and strengths of the review

The pooled estimates of this review are from moderately heterogeneous primary studies. The use of wide inclusion criteria for the review might have contributed to the observed moderate level of heterogeneity. Indeed, attempts were made to overcome the concern of heterogeneity by using the random effects model, although it still results in a less precise estimate or wide confidence interval. The subgroup analyses also contributed to addressing it to some extent. However, due to the unavailability of potential factors from primary studies or because of the unmeasured variables that could affect EHR or EMR's effect on mortality, we could not sufficiently minimize the heterogeneity. In addition, this review was limited to studies published in English. This may have introduced English language bias.

## Conclusion

The review revealed that the implementation of electronic records in inpatient, specialized and intensive care units, and primary health care settings seems to result in a statistically significant reduction in mortality, despite moderate heterogeneity among the studies. It also shows that the effect of electronic records on mortality may be insignificant or negative at the early phase of the implementation, and then progressively improves to reduce mortality at the later stages of maturation.

Evidence from this review shows that meaningful use of electronic records reduces mortality. The features embedded in and the maturation of the electronic health records are important elements influencing the mortality.

## Data Availability

The raw data supporting the conclusions of this article will be made available by the authors, without undue reservation.

## Supplementary material

Supplementary Material A Full search strategy.

Supplementary Material B Protocol for the systematic review and meta-analysis.

Supplementary Material C Meta-data.
