# Supplementary material for: Effect of electronic records on mortality among patients in hospital and primary healthcare settings: a systematic review and meta-analyses
Source: Front Digit Health. 2024 Jun 26;6:1377826. doi: 10.3389/fdgth.2024.1377826 (PMC11233798; doi:10.3389/fdgth.2024.1377826)
Supplement: Supplementary Material A — Full search strategy. [file Table1.docx]

**Full search strategy**

1. **PubMed**
2. "EMR"[ti] OR "Electronic medical record*"[ti] OR "Electronic health record*"[ti] OR "EHR"[ti] OR “Hospital information systems” [ti] OR “Medical records linkage” [ti] OR “Decision support systems” [ti] OR “Personal health record*”[ti] OR “Computerized provider order entry” [ti] OR “Computerized Physician Order Entry” [ti] OR “Clinical information system*”[ti] OR “Electronic Patient Record*”[ti] OR "Electronic health record*"[Mesh]
3. Effect[ti] OR Predict*[ti] OR Association[ti] OR Compar*[ti] OR Value[ti] OR Impact[ti] OR Benefit[ti] OR Improvement[ti]
4. Quality[ti] OR Efficiency[ti] OR Risk[ti] OR Safety[ti]
5. #2 OR #3
6. Mortality[ti] OR Morbidity[ti] OR “Healthcare outcomes” [ti] OR “Health care outcomes”[ti] OR “Health outcomes”[ti] OR Admission[ti] OR Readmission[ti] OR Hospitalization[ti] OR Rehospitalization[ti] OR "Hospital length of stay"[ti] OR "Length of stay"[ti] OR "Health service quality"[ti] OR "Service quality"[t]OR Mortality[Mesh] OR Morbidity[Mesh]
7. #1 AND #4 AND #5
8. **SCOPUS**

TITLE-ABS((Effect OR Impact) AND (EMR OR "Electronic medical record*" OR "Electronic health record*" OR EHR) AND (Mortality OR Morbidity OR "Healthcare outcomes" OR "Health care outcomes" OR "Health outcomes") )

1. **EMBASE**

(emr:ti,ab,kw OR 'electronic medical record*':ti,ab,kw OR 'electronic health record*':ti,ab,kw OR ehr:ti,ab,kw) AND (mortality:ti,ab,kw OR morbidity:ti,ab,kw OR 'healthcare outcomes':ti,ab,kw OR 'health care outcomes':ti,ab,kw OR 'health outcomes':ti,ab,kw) AND (effect:ti,ab,kw OR impact:ti,ab,kw)

1. **CINAHL**

**Concept-1**

(MH “Electronic Health Record”) OR “electronic health record*” OR “EHR” OR “Electronic Medical record*” OR “EMR” OR “electronic patient record*” OR (MH “Medical Records, Personal“) OR (MH “Computerized Decision Support System”) OR “computerized decision support system” OR “CDSS” OR (MH “Computerized Physician Order Entry”) OR “computerized physician order entry” records system*” OR “CPOE” OR (MH “Computerized medical records system*” OR “Computerized health records system*” OR “Patient Record System*”

**Concept-2**

“Mortality” OR “Morbidity” OR (MH “Healthcare outcomes”) OR “Health care outcomes” OR “Health outcomes” OR (MH “Admission”) OR “Readmission” OR “Hospitalization” OR (MH "Hospital length of stay") OR "Length of stay" OR (MH "Health service quality") OR "Service quality"

1. **Cochrane**

#1 MeSH descriptor: [Electronic Health Records] explode all trees

#2 (Electronic NEXT medical NEXT record*):ti,ab,kw OR (Electronic NEXT health NXT record*):ti,ab,kw OR (EHR):ti,ab,kw OR (EMR):ti,ab,kw (Word variations have been searched)

#3 #1 OR #2

#4 (Mortality):ti,ab,kw OR (Morbidity):ti,ab,kw OR (Healthcare NEXT outcome*):ti,ab,kw OR (Health NEXT care NEXT outcome*):ti,ab,kw OR (Health outcome*):ti,ab,kw (Word variations have been searched

#5 #3 AND #4

1. **Google scholar**

**With all of the words:** Effect "Electronic medical record*" Mortality

**With the exact phrase:** "Electronic medical record*" "Electronic health record*" "Healthcare outcomes" "Health care outcomes"

**With at least one of the words:** Effect Impact "Electronic medical record*" EMR "Electronic health record*" EHR Mortality Morbidity "Healthcare outcomes" "Health care outcomes"
