# Supplementary material for: Effect of electronic records on mortality among patients in hospital and primary healthcare settings: a systematic review and meta-analyses
Source: Front Digit Health. 2024 Jun 26;6:1377826. doi: 10.3389/fdgth.2024.1377826 (PMC11233798; doi:10.3389/fdgth.2024.1377826)
Supplement: Supplementary Material C — Meta-data. [file Datasheet1.pdf]

Effect of electronic medical and health records on mortality: a systematic review and meta-analysis

To enable PROSPERO to focus on COVID-19 submissions, this registration record has undergone basic automated checks for eligibility and is published exactly as submitted. PROSPERO has never provided peer review, and usual checking by the PROSPERO team does not endorse content. Therefore, automatically published records should be treated as any other PROSPERO registration. Further detail is provided here.

Citation

Lemma Derseh Gezie, Tariku Nigatu Bogale. Effect of electronic medical and health records on mortality: a systematic review and meta-analysis. PROSPERO 2023 CRD42023437257 Available from:
https://www.crd.york.ac.uk/prospero/display_record.php?ID=CRD42023437257

Review question

Does Electronic Health records and electronic medical records have effect on mortality

Searches

1. Cochrane

2. MEDLINE/Ovid

3. EMBASE

4. Scopus

5. Google Scholar (for gray literature too)

Published in English, no limit in Publication year, no restriction in study design

Types of study to be included

Both observational and interventional studies. RCT, cohort (prospective/retrospective), case-control, and cross-sectional, or any related analytic designs.

Condition or domain being studied

Mortality

Participants/population

Papers that studied the following population will be considered eligible: people with any health condition who visited health facilities, any age, any gender, any level of disease severity, and studies conducted anywhere across the globe.

Intervention(s), exposure(s)

The review considers papers that examined people who were treated in facilities where EMR or EHR are implemented; it also includes papers that studied settings where routine data utilization is commonly practiced for any healthcare related

decisions.

Comparator(s)/control

People who have been admitted to hospitals/facilities in which EMR or HER are not implemented are considered as control group. For the second objective, the control group will be health professionals (or the corresponding patients) who usually do not make decisions based on or by reviewing currently available evidences, but with their own prior knowledge and experiences.

Context

Generally, the whole review process will strictly follow the preferred reporting items for systematic review and meta-analysis (PRISMA) guidelines. Thus, studies will be considered eligible if they fulfill the PICO (Population, Intervention/Exposure, Comparison, and Outcome) criteria:

The eligible study settings will be hospitals or health facilities in which EMR and EHR are implemented. Studies will be also considered eligible if they investigated the association between the EMR implementation and mortality by comparing with a control group who did not use the EMR or EHR (or do not use evidence for routine activities). There will be no limit on the publication status and date of publication though it is required to be written in English. However, we will only consider studies conducted exclusively in humans.

The review excludes studies that do not have both the intervention/exposure and control groups, are case reports or case series, and if the full document is not available.

Main outcome(s)

Mortality

Measures of effect

RR, OR, Beta-coefficient, and percent,

Additional outcome(s)

No other outcome variable is our interest

Data extraction (selection and coding)

In the review process, there will be three reviewers in total. Two of the reviewers will independently review the articles, and the third one will resolve disagreements if any. However, first, we will agree on the data elements to be extracted as well as on the keywords and MeSH terms that will be used in the search process.

Two reviewers LD and TN will independently extract data from all eligible studies using the data extraction checklist from experimental and observational studies. If there is any disagreements, it will be discussed with a third reviewer (TA) and resolved by consensus. Data items will be captured in Microsoft Excel spreadsheets. Thus, the following general data items will be extracted from each study: the first author’s name, year of publication, geographical region, country, sample size, study design, and method of analysis. The outcome variable or mortality will be also extracted.

Moreover, study specific characteristics such as data related to the baseline characteristics of cohort study participants, and the duration of follow-up will also be recorded. Moreover, we will extract data on which covariates were adjusted for in the regression models of the included studies, and report on the measurement of potential confounders.

Different papers report their findings in a different format and as a result, the results may not be suitable for meta-analysis. In other words, the effect size, say θ and its standard error (SE) may not be readily available in some studies.

Therefore, so as not to exclude these papers or the data elements unnecessarily, we will extract the data that can be transformed into the required format for meta-analysis, i.e. effect size and its SE (or θ and SE (θ)).

Risk of bias (quality) assessment

The identified studies that meet all the inclusion criteria mentioned above and from which data were extracted will be grouped in either of the following designs: experimental studies, cohort studies, and case-control studies. Then by using the respective quality assessment tool for each study design, the studies will be assessed independently by two reviewers for methodological validity. This will be done by using the tools developed by the Juana Briggs Institute (JBI) from the SUMARI suite (9). Any disagreements that arise between the reviewers will be resolved through discussion and with the assistance of a third reviewer when required. In addition, we will involve content experts to judge any other flaws that could be overlooked by non-experts. After the appraisal, to manage the risk of bias, we will consider all studies for the review unless there is an obvious reason to reject it; however, we may conduct subgroup analysis by the quality of studies (low, medium, and high) later. Finally, Kappa statistics will be determined to measure the level of agreement between the two reviewers before it was resolved by discussion or the third reviewer, and then it will be reported in the manuscripts.

Strategy for data synthesis

Before conducting any quantitative analysis, first, the extracted data will be examined to check whether it is possible to conduct a meta-analysis (pooling of estimates) on it or not. For those data elements on which we cannot conduct a meta-analysis (after making all the efforts described above), we will conduct only narrative synthesis. However, those studies on which we will conduct meta-analysis will also be used for narrative synthesis. In this regard, first we will present the characteristics of primary studies such as the primary authors, year of publication, country and region in which the study was conducted, its study design, sample size, participants, etc. to preset the basic features of primary studies. Then we will narrate the findings related to the research questions including mortality, cure rate, time spent for documentation, guideline adherence, medication error, adverse drug effects, cost of healthcare, and so on.

Thus, the narrative synthesis will be done alongside (mixed with) the meta-analysis and will be carried out using a framework that consists of four elements:

o Developing a theory of how EMR works, why, and for whom

o Developing a preliminary synthesis of findings of included studies

o Exploring relationships within and between studies

o Assessing the robustness of the synthesis

Thus, we will synthesize narratively the prevalence of and different effect sizes related to the effect of EMR on healthcare outcomes, and present by different characteristics such as by country, geographic region, and study design. Measures of association such as the odds ratio (OR), risk ratio (RR), and hazard ratios, and beta coefficients will be synthesized as per their suitability. Similar analyses will be conducted to determine the effect of data use on the healthcare outcomes. In all these analysis, the focus will be to identify the pattern of relationships (if any), especially if the effect sizes differ by the categories of potential confounders or contextual variables. Thus, relationships among variables will be identified for both EMR and use of data on healthcare outcomes.

Analysis of subgroups or subsets

In the presence of considerable heterogeneity, we will try to address it with different techniques. For example, to account for the heterogeneity due to differences in contexts in which the primary studies were conducted, we will apply random effect model than using the fixed effect model. Using the random effect model would help us to capture the heterogeneity of estimates which otherwise inflates their variability that would in turn compromise the reliability of pooled estimates.

In addition, we will conduct subgroup analysis to measure the effect of EMR by the categories of potential confounders such as by geographic region, development status of the country/region in which the primary study was conducted (low,

middle, and high income countries), study design, quality of the studies as per the appraisal result, and other characteristics. The statistical methods such as the Cochrane Q-statistics and I^2 value will also be determined to check whether the problem of heterogeneity is addressed with the subgroup analysis. If the subgroup analyses address the observed heterogeneity, we will report the pooled estimate and plausible justifications will be inquired and discussed with the literature and theory.

Contact details for further information

Lemma Derseh Gezie

lemmagezie@gmail.com

Organisational affiliation of the review

University of Gondar

Review team members and their organisational affiliations

Dr Lemma Derseh Gezie. University of Gondar

Tariku Nigatu Bogale. JSI

Type and method of review

Epidemiologic, Intervention, Meta-analysis, Narrative synthesis, Systematic review

Anticipated or actual start date

18 June 2023

Anticipated completion date

28 July 2023

Funding sources/sponsors

JSI

Conflicts of interest

Language

English

Country

Ethiopia

Stage of review

Review Ongoing

Subject index terms status

Subject indexing assigned by CRD

Subject index terms

Electronic Health Records; Electronics, Medical; Humans

Date of registration in PROSPERO

27 June 2023

Date of first submission

16 June 2023

Stage of review at time of this submission

The review has not started

Stage	Started	Completed
Preliminary searches	No	No
Piloting of the study selection process	No	No
Formal screening of search results against eligibility criteria	No	No
Data extraction	No	No
Risk of bias (quality) assessment	No	No
Data analysis	No	No

The record owner confirms that the information they have supplied for this submission is accurate and complete and they understand that deliberate provision of inaccurate information or omission of data may be construed as scientific misconduct.

The record owner confirms that they will update the status of the review when it is completed and will add publication details in due course.

Versions

27 June 2023

27 June 2023
